# Identification of health-related behavioural clusters and their association with demographic characteristics in Irish university students

**DOI:** 10.1186/s12889-019-6453-6

**Published:** 2019-01-28

**Authors:** Joseph J. Murphy, Ciaran MacDonncha, Marie H. Murphy, Niamh Murphy, Anna Timperio, Rebecca M Leech, Catherine B. Woods

**Affiliations:** 1 Department of Physical Education and Sport Sciences, University of Limerick, Castletroy, Limerick, Ireland; 2 Health Research Institiute, University of Limerick, Limerick, Ireland; 3 School of Sport, Ulster University, Antrim, UK; 4 Department of Health, Sport and Exercise Science, Waterford Institute of Technology, Waterford, Ireland; 5 Institute of Physical Activity and Nutrition, School of Exercise and Nutrition Sciences, Deakin University, Geelong, Australia

**Keywords:** University students, Health-related behaviours, Clustering

## Abstract

**Background:**

Students engage in risky health-related behaviours that influence their current and future health status. Health-related behaviours cluster among adults and differently based on sub-populations characteristics but research is lacking for university populations. Examining the clustering of health- related behaviours can inform our initiatives and strategies, while examining cluster members’ characteristics can help target those who can prosper most from health promotion efforts. This study examines the clustering of health-related behaviours in Irish university students, and investigates the relationship with students’ sex, age, field of study and accommodation type.

**Methods:**

An online survey was completed by 5672 Irish university students (51.3% male; 21.60 ± 5.65 years) during 2014. Two-step cluster analysis was used to understand how health-related behaviours (physical activity, smoking, alcohol intake, drug use and dietary habits) cluster among male and female students. Binary logistic regressions were conducted to examine the likelihood of students falling into certain clusters based on their characteristics.

**Results:**

Five cluster groups were identified in males and four in females. A quarter of males were categorised as ideal healthy with older students and those from certain fields of study having a higher likelihood of being classified in a low physical activity and poor diet (OR = 1.06–2.89), alcohol consumption (OR = 1.03–3.04), or smoking and drug use (OR = 1.06–2.73) cluster. Forty-five percent of females were categorised as ideal healthy with older females more likely to be in a low active and smoking cluster (OR = 1.03), and less likely to be in a convenience food cluster (OR = 0.96). Females from certain fields of study were also more likely to be classified in these clusters (OR = 1.59–1.76). Students living away from their family home had in increased likelihood of being in a cluster related to a higher frequency of alcohol consumption (OR = 1.72–3.05).

**Conclusion:**

Health-related behaviours cluster among this population and need to be taken into account when designing multi-health interventions and policies. These findings can be used to target student groups at risk, leading to more efficient and successful health promotion efforts. The addition of modules providing information regarding health-related behaviours are advised in all fields of study.

## Background

Unhealthy or risky health-related behaviours (HRB) are primary causes of premature morbidity and mortality [1–3]. Physical inactivity, alcohol abuse, smoking tobacco, and poor dietary behaviours are suggested as the four main contributors to diseases such as hypertension, diabetes and certain cancer [1]. There is a risk that individuals will engage in risky HRB such as above, including illicit drug use, as they gain increased independence when they transition from high school to university [4, 5]. A recent study found that 36% of Irish university students (*n* = 8122; 49.1% male; 23.17 ± 6.75 years) reported being insufficiently active, while 22% drank alcohol at least twice a week, 21% smoke tobacco (occasionally or frequently), and 20% had previous or current illicit drug use [6]. Earlier studies have also shown the high proportion of university populations engaging in risky HRB, which is worrying. Two thirds of Irish undergraduate students (*n* = 2250; 36.9% male) indicated hazardous alcohol consumption [7], with another study finding that a quarter of Irish students were smokers and 37% had used illicit drugs in the past year [8]. Much like with physical activity (PA), some students also fail to maintain healthy dietary behaviours, which are associated with reduced risk of developing chronic diseases [4, 9, 10].

Studies often examine HRB in isolation of one another [5, 7, 11], but emerging research suggests that these behaviours co-exist or cluster in most populations [3]. While associations between risky HRB have been noted [5], little is known about the clustering of these behaviours nor their relationship with demographic (i.e. sex, age) and environmental (i.e. living accommodation) factors in this population [2, 4, 12, 13]. Noble and colleagues (2015) conducted a review of behavioural cluster research, identifying 56 relevant articles in adult populations. The most popular cluster groups reported were a healthy cluster containing no risk factors (81%), smoking and alcohol consumption (56%), all risky health behaviours (i.e. physical inactivity, alcohol consumption, smoking, and poor dietary behaviours; 50%), and poor diet with physical inactivity (44%). Four of the articles identified in this review included university populations from the UK and the USA [1, 14–16], with one paper examining the general Irish population [17]. In total, the studies examining university populations looked at 5585 students, age ranges from 18 to 24 years, with three studies looking at mixed sex populations and one looking at females only. The clusters that emerged in each study fall somewhere on a scale from unhealthy/ high-risk clusters to moderate-risk and low-risk for health. Effective interventions that lead to the adoption of healthy behaviours and to the reduction of risky behaviours are needed [10]. There is currently a gap in our understanding of if and how these behaviours cluster and co-exist in this population, which can help target and tailor future health promotion efforts and increase their success.

The clustering of HRB allows us to study how groups of students engage in a range of behaviours, but few studies have explored whether specific sub-groups of populations are more or less likely to be classified in certain clusters [2, 18]. Research has shown that males and younger age groups are associated with more risky health cluster combinations [3]. Although this is useful, we have yet to investigate how a students’ accommodation type or field of study associates with possible clusters of HRB. Living environment is a factor worth considering when examining HRB, whereby some students live in their family home while others live in student or rented accommodation [19, 20]. Students living in university accommodation report higher PA levels [20, 21] but have risky dietary habits [22] and an increased prevalence of binge drinking [23, 24] when compared to those living off university campuses. Risky dietary habits were seen as the increased consumption of convenience foods such as snacks, sweets, cakes and fast foods [22]. Behavioural differences have also been observed between students studying in different fields, with biological students showing increased alcohol consumption, arts and social science students more likely to smoke and use illicit drugs [25], and students studying sport and health-related courses less likely to display poor dietary behaviours [26]. These young adults are in a learning environment and are still at an age where HRB that influence future health status can be influenced and directed [10], but research has shown the inefficiency of health behaviour strategies with a one -for-all approach [27]. Examining the characteristics of cluster members will help to identify students who express similar HRB, therefore, identifying potential target groups for health promotion efforts [18]. Thus, the purpose of this study is to investigate the clustering of HRB in Irish university students and identify student types classified within these clusters.

## Methods

Data for this study was collected during October and November 2014 as part of the Student Activity and Sport Study Ireland (SASSI) [6]. Due to the all-island approach, the permission to conduct the study was granted from relevant ethical committees in the Republic of Ireland (Waterford Institute of Technology School of Health Science Research Ethics Committee; Dublin City University Research Ethics Committee), Northern Ireland (Ulster University Research Governance), and extended through recognition by all institutes involved. Participants (*N* = 9197) from 31 institutes of higher education around Ireland using quota based sampling considering institution size and field of study were administered the survey, while 8122 (50.9% male; 21.51 ± 5.55 years) of those completed the minimum amount required for analysis (i.e. demographic data). Relevant personnel (e.g. health promotion officer, health lectures, and health researchers) volunteered in each of the universities to recruit students and administer the survey via a Survey Monkey link during class time. Recruitment was done through emails and direct contact with lecturers and heads of departments to allow access to the class groups required based on the sampling provided. Weekly updates were provided to the relevant personnel in each university to ensure that the correct students were being administered the survey. The survey could be accessed through any smartphone, laptop or computer. The use of an online survey administered in a class, instead of through emails, was based on previous research protocols where participation rates in excess of 90% were achieved [28]. Participation in the online survey was voluntary and anonymous. Students were informed that by completing the questionnaire they were providing their informed consent to participate. They were also informed that they could exit the survey at any time by closing the survey window on their smartphone, laptop or computer. The survey included study information, demographic information (age, sex, field of study and accommodation type), PA levels, risky HRB, and dietary habit questions. Field of study was grouped as relating to i) social, business and law; ii) health, welfare and exercise; iii) humanities and arts; iv) education; v) science, maths and computing; vi) engineering and manufacturing; vii) other. Accommodation was either recognised as living in a family home or living outside of the family home (e.g. student accommodation, renting privately etc.).

Participants PA levels over the last 7 days was measured using the International Physical Activity Questionnaire – Short Form (IPAQ – SF) [29]. Participants were classified into ‘low’, ‘moderate’ and ‘high’ categories depending on the level of PA reported from the nine items. Various interpretations of IPAQ can be used, but for the purpose of this study it was considered that only those categorised as ‘high’ were meeting the physical activity guidelines (PAGL) [30]. Participants were then dichotomised into meeting or not meeting the PAGL. This was based on the current PAGL, which state that adults should engage in at least 150 min of moderate-intensity aerobic PA, or 75 min of vigorous-intensity aerobic PA in bouts of at least 10-min each week [31]. The IPAQ – SF has previously been found to have acceptable validity and reliability in university students [32, 33]. Items assessing smoking, drug use, alcohol consumption and dietary habits were taken from the Survey of Lifestyle and Attitudes to Nutrition (SLÁN) study [34]. Smoking levels were assessed using a single item: ‘Do you now smoke every day, some days or not at all?’ Answers were dichotomised into yes (yes and sometimes) and no. Drug use was assessed using a single item: ‘Have you ever taken non-prescribed/recreational drugs?’ Answers were dichotomised into any drug use (yes and yes but have stopped) and no previous or current drug use. Alcohol consumption was assessed using a single item: ‘How often do you have a drink containing alcohol?’ Answers were dichotomised into % ≤2 times/week (never, monthly or less and 2–4 times a month) and % ≥2 times/week (2–3 times a week and 4+ times a week). Dietary habits were assessed by asking first ‘How often do you eat convenience food (i.e. fast food, takeaways, Chinese, Indian, burgers, chips etc.)’ where the answers were dichotomised into % ≤ once/week (never or less than once a week) and % ≥ once/week (1–3 times a week, 4–6 times a week or daily). A second question asked ‘How often do you prepare food from fresh ingredients rather than pre-prepared food?’ where the answers were dichotomised into % ≤4 times/week (never, less than once a week or 1–3 times a week) and % ≥4 times/week (4–6 times a week or daily). Although the measures in this study were different, dichotomisation of responses was based on a similar approach used in the research [18]. Consequently, this also aided with the interpretation of cluster outputs.

### Statistical analysis

SPSS Inc., Chicago IL, version 23 was used for all analyses. Participants who did not complete all of the items needed for the cluster analysis were removed from the study. Descriptive statistics were calculated for demographic data and each of the behaviours. Pearson’s Chi-square test for independence was performed to note any significant differences for each behaviour between sexes. Engagement in HRB was different for males and females [35–38] and previous studies have investigated the clustering of behaviours separately based on sex [14, 15]. Thus, a two-step cluster analysis was used as an explanatory tool to identify specific behavioural clusters in male and female students separately. This method was designed to handle large data sets and enables the input of categorical variables [39]. The number of clusters was based on the log-likelihood distance and Schwarz Bayesian criterion [39]. The cluster analysis procedures were repeated in an internal random sample of 50% of the total study sample for each sex and a kappa statistic was used to assess reliability of the cluster solutions [40]. The cluster outputs were given descriptive names based on the behaviours evident in each one. An ANOVA with Bonferroni (or Games-Howell when lack of homogeneity) post hoc was used to test the difference between clusters for mean age. Pearson’s Chi square was assessed to test for differences in student characteristics (field of study and accommodation type) between the clusters. In order to assess the number of students from certain fields of study and accommodation types falling into each cluster, the adjusted residual (AR) was observed [41]. When the AR rises above 2.0 it is presumed that a significantly higher proportion of students are in a certain cluster than what is expected. When the adjusted residual falls below − 2.0 it is presumed that a significantly lower proportion of students are in a certain cluster than what is expected [41]. Further analysis was completed using binary logistic regressions to identify those students who had a higher likelihood of being categorised in a cluster containing a risky HRB. The health, welfare and exercise students and the students living in their family home were used as the reference categories since they are seen to engage in less risky HRB [22–24, 26]. Results are presented as Odds Ratios (OR) and 95% Confidence Intervals (CI).

## Results

After data cleaning, the analytical sample comprised of 5672 participants (51.3% male; 21.60 ± 5.65 years). The final sample were older (t (7611) = 2.30, *p* < 0.05) than those excluded, with no difference for sex (X^2^ = 0.57 (1), *p* = 0.33). Baseline characteristics for the study sample are shown below (Table 1). Compared to females, a higher proportion of male students met the PAGL, had a higher frequency of alcohol consumption, smoked, reported current or past drug use, and consumed convenience foods more/ than once a week. A higher proportion of females reported consuming fresh food at least four times a week.

**Table 1:** Baseline characteristics of the study sample

	Total (%) (*n* = 5672)	Males (%) (*n* = 2907)	Females (%) (*n* = 2765)
Student Characteristics			
*Student Type (%)*			
Undergraduate	95.1	95.6	94.5
Postgraduate	4.9	4.4	5.5
Full-time	95.5	96.0	95.0
Part-time	4.5	4.0	5.0
*Student Field of Study (%)*			
Science, maths and computing	29.0	39.8	17.5
Social, business, law and tourism	21.5	18.0	25.2
Health, welfare and exercise related	18.2	16.5	20.0
Humanities and arts	14.1	9.6	18.9
Education	6.5	2.9	10.3
Engineering and construction	5.4	8.6	2.1
Other courses	5.3	4.6	6.0
*Accommodation Type (%)*			
At home	48.1	52.1	44.0
Away from home	51.9	47.9	56.0
Behavioural Characteristics			
Physical Activity: Meeting Guidelines**	66.0	72.6	59.0
Alcohol: % ≥2 times/week**	22.6	25.1	20.0
Smoking: % Yes**	19.3	21.2	17.4
Drug Use: % Yes**	19.0	26.5	11.1
Diet (Convenience): % ≥ once/week**	39.5	44.2	34.7
Diet (Fresh food prep): % ≥4 times/week**	58.4	55.5	61.4

**= significant difference between male and female responses (*p* < 0.01)

### Cluster outputs and characteristics

Cluster analysis revealed five distinct clusters for males and four for females. There was a very good agreement between the cluster solution derived from the full sample and the random subsample (50%; males: kappa = 0.82, *p* < 0.01; females: kappa = 1.00, p < 0.01). The distribution of behaviours (i.e. characteristics) within each cluster is shown for both male and female students (Table 2). For example, Cluster 1 in males, labelled ‘Ideal Healthy’, was characterised by meeting the PAGL, low risk relating to smoking, drug use, frequency of alcohol and convenience food consumption, and the highest proportion of students that prepared food using fresh ingredients. For females, the ‘Ideal Healthy’ cluster had similar behaviours identified, except the proportion meeting the PAGL was lower (63.4% vs 100.0%) and the proportion consuming fresh foods at least four times a week was higher (69.6% vs 65.1%). Other clusters saw engagement in a range of behaviours but they were given a descriptive name based on any predominant risky HRB.

**Table 2:** Cluster outputs and characteristics in male and female students according to the health-related behaviours assessed

Male Students						
Cluster Number		1	2	3	4	5
Cluster Title		Ideal Healthy	Low PA & Poor Diet	Convenience Food	Alcohol Consumption	Smoking & Drug Use
N (%)		720 (24.8%)	635 (21.8%)	441 (15.2%)	476 (16.4%)	635 (21.8%)
Characteristics						
		(%)	(%)	(%)	(%)	(%)
PAGL	Meeting	100.0%	0.0%	100%	66.0%	100.0%
Alcohol Intake	≥2times/week	0.0%	10.7%	0.0%	100.0%	29.4%
Smoking	Yes	0.0%	23.9%	0.0%	12.2%	63.8%
Drug Usage	Any Use	0.0%	30.1%	0.0%	20.6%	75.6%
Convenience	≥once/week	0.0%	51.8%	100.0%	43.1%	48.7%
Fresh Food	≥4times/week	65.1%	43.8%	50.3%	52.3%	62.2%
Female Students						
Cluster Number		1	2	3	4	
Cluster Title		Ideal Healthy	Low PA & Smoking	Convenience Food	Alcohol Consumption	
N (%)		1246 (45.1%)	632 (22.8%)	541 (19.6%)	346 (12.5%)	
Characteristics						
		(%)	(%)	(%)	(%)	
PAGL	Meeting	62.4%	50.0%	60.4%	61.0%	
Alcohol Intake	≥2times/week	0.0%	32.8%	0.0%	100.0%	
Smoking	Yes	0.0%	75.9%	0.0%	0.0%	
Drug Usage	Any Use	0.0%	48.6%	0.0%	0.0%	
Convenience	≥once/week	0.0%	42.2%	100.0%	43.6%	
Fresh Food	≥4times/week	69.6%	57.6%	50.6%	55.8%	

### Proportion of students categorised in each cluster

The differences between clusters based on age, field of study and accommodation type can be seen for male and female students (Table 3). Compared to the ‘Ideal Healthy’ clusters, males were significantly older in the ‘Low PA & Poor Diet’ and ‘Smoking & Drug Use’ clusters, while females were older in the ‘Low PA & Smoking’ cluster and younger in the ‘Convenience Food’ cluster. A Pearson Chi-square showed a significant difference between the students’ field of study and cluster placement for males and females. A Pearson Chi-square also identified differences between the students’ accommodation type and their cluster placement in males and females.

**Table 3:** Proportion of students falling into each cluster, according to age, field of study and accommodation type

Male Students					
Cluster Title	Ideal Healthy	Low PA & Poor Diet	Convenience Food	Alcohol Consumption	Smoking & Drug Use
Age (Mean ± SD)	21.05 ± 5.00	22.80 ± 6.85**	20.80 ± 4.70	21.57 ± 5.84	22.25 ± 5.24**
*Field of Study* - n (%)					
Health, Welfare & Exercise	162 (22.7)^a^	70 (11.2)^b^	74 (17.1)	63 (13.4)	104 (16.7)
Social, Business, Law	107 (15.0)^b^	107 (17.1)	74 (17.1)	106 (22.5)^a^	123 (19.7)
Humanities & Arts	55 (7.7)^b^	53 (8.5)	36 (8.3)	53 (11.3)	79 (12.7)^a^
Education	27 (3.8)	16 (2.6)	20 (4.6)^a^	10 (2.1)	10 (1.6)^b^
Science, Maths, Computing	273 (38.2)	292 (46.7)^a^	174 (40.1)	176 (37.4)	226 (36.2)^b^
Engineering & Manufacturing	62 (8.7)	56 (9.0)	37 (8.5)	36 (7.6)	54 (8.7)
Other	28 (3.9)	31 (5.0)	19 (4.4)	27 (5.7)	28 (4.5)
*Accommodation* - n (%)					
At home	392 (55.8)^a^	362 (58.0)^a^	256 (58.9)^a^	148 (31.5)^b^	328 (52.7)
Away from home	310 (44.2)^b^	262 (42.0)^b^	179 (41.1)^b^	322 (68.5)^a^	294 (47.3)
Female Students					
Cluster Title	Ideal Healthy	Low PA & Smoking	Convenience Foods	Alcohol Consumption	
Age (Mean ± SD)	21.41 ± 5.62	22.52 ± 6.50**	20.58 ± 3.71**	21.00 ± 6.23	
*Field of Study* - n (%)					
Health, Welfare & Exercise	274 (22.2)^a^	100 (16.1)^b^	101 (19.1)	70 (20.4)	
Social, Business, Law	267 (21.7)^b^	179 (28.7)^a^	146 (27.7)	95 (27.7)	
Humanities & Arts	209 (17.0)^b^	146 (23.3)^a^	95 (18.0)	64 (18.7)	
Education	169 (13.7)^a^	23 (3.7)^b^	50 (9.5)	40 (11.7)	
Science, Maths, Computing	205 (16.6)	124 (19.9)	95 (18.0)	55 (16.0)	
Engineering & Manufacturing	26 (2.1)	11 (1.8)	13 (2.5)	6 (1.7)	
Other	82 (6.7)	40 (6.4)	28 (5.3)	13 (3.8)	
*Accommodation* - n (%)					
At home	539 (43.9)	275 (44.1)	278 (52.1)^a^	107 (31.7)^b^	
Away from home	690 (56.1)	348 (55.9)	256 (47.9)^b^	231 (68.3)^a^	

ANOVA: ** = < 0.01; Adjusted Residuals: ^a^ = > 2.0 or significantly higher proportion than expected falling into this cluster, ^b^ = < 2.0 or significantly fewer proportion than expected falling into this cluster. Numbers are reduced due to missing data for demographic data

For males the ‘Ideal Healthy’ cluster contains a significantly higher proportion of students from health, welfare and exercise related courses (AR = 5.15) and living in their family home (AR = 2.29). A significantly lower proportion of social, business and law students (AR = − 2.44), and humanities and arts students (AR = − 2.01) were seen in this cluster. A ‘Low PA & Poor Diet’ cluster contains a higher proportion of students from science, maths and computing related courses (AR = 4.01) and living in their family home (AR = 3.35). This cluster contains a lower proportion of health, welfare and exercise related students (AR = − 4.03). The ‘Convenience Food’ cluster contains a higher proportion of students from education related courses (AR = 2.31) and living in their family home (AR = 3.07). An ‘Alcohol Consumption’ cluster contains a higher proportion of students from social, business and law courses (AR = 2.76) and living away from their family home (AR = 9.78). The ‘Smoking & Drug Use’ cluster contains a higher proportion of humanities and arts students (AR = 2.91) but a lower proportion of education (AR = − 2.18) and science, maths and computing related students (AR = − 2.06).

For females, the ‘Ideal Healthy’ cluster contains a significantly higher proportion of health, welfare and exercise related (AR = 2.66) and education related students (AR = 5.25). A lower proportion of students from social, business and law related courses (AR = − 3.85) and humanities and arts (AR = − 2.29) were classified in this cluster. A ‘Low PA & Smoking’ cluster contains a higher proportion of social, business and law related (AR = 2.31) and humanities and arts students (AR = 3.33). This cluster also contains a lower proportion of health, welfare and exercise related (AR = − 2.80) and education (AR = − 6.21) students. The ‘Convenience Foods’ cluster contains a higher proportion of students living in their family home (AR = 4.18) while the ‘Alcohol Consumption’ cluster contains students living away from their family home (AR = 4.89).

### Likelihood of students being categorised in each cluster

Binary logistic regressions revealed the students with a higher likelihood of falling into clusters containing risky HRB, when compared to the ‘Ideal Healthy’ cluster for males and females (Table 4). Cluster placement was considered as the dependent variable while students’ age, field of study and accommodation type were seen as the independent variables. For males, the regression models were significant for the ‘Low PA & Poor Diet’ (X^2^(8) = 67.891, *p* < 0.01; R^2^ = 7.2%), ‘Alcohol Consumption’ (X^2^(8) = 107.451, p < 0.01; R^2^ = 12.8%) and ‘Smoking & Drug Use’ (X^2^(8) = 52.414, p < 0.01; R^2^ = 5.6%) clusters. A one year increase in age increased the likelihood of males being classified in the ‘Low Active & Poor Diet’ (OR = 1.06, CI = 1.04–1.09), ‘Alcohol Consumption’ (OR = 1.03, CI = 1.00–1.05), and ‘Smoking & Drug Use’ (OR = 1.06, CI = 1.03–1.08) clusters. Males from all fields of study, except education had a higher likelihood of falling into the ‘Low PA & Poor Diet’ cluster (OR = 2.03–2.89, CI = 1.25–5.27) when compared to the health, welfare and exercise students. Those from all fields of study, except education and engineering and manufacturing had a higher likelihood of falling into the ‘Alcohol Consumption’ cluster (OR = 1.81–3.04, CI = 1.25–5.52). Males studying in social business and law (OR = 2.10, CI = 1.43–3.07), humanities and arts (OR = 2.72, CI = 1.74–4.27), and science and computing (OR = 1.46, CI = 1.06–2.02) related courses had a higher likelihood of being categorised in the ‘Smoking & Drug Use’ cluster, when compared to the reference category. Males living away from home had an increased likelihood of being classified in the ‘Alcohol Consumption’ (OR = 3.05, CI = 2.34–3.98) cluster.

**Table 4:** Likelihood of students being classified in a cluster containing a risky health behaviour based on their age, field of study and accommodation type

Male Students								
Cluster Title	Low PA & Poor Diet		Convenience Food		Alcohol Consumption		Smoking & Drug Use	
N	581		406		431		569	
Participant Demographics								
	OR	95% CI	OR	95% CI	OR	95% CI	OR	95% CI
Age *(years)*	1.06**	1.04–1.09	0.99	0.96–1.02	1.03*	1.00–1.05	1.06**	1.03–1.08
Field of Study *(Reference Cluster = Health, Welfare & Exercise)*								
Social, Business, Law	2.34**	1.55–3.54	1.65*	1.08–2.51	3.00**	1.96–4.60	2.10**	1.43–3.07
Humanities & Arts	2.08**	1.25–3.45	1.57	0.93–2.65	3.04**	1.80–5.13	2.73**	1.74–4.27
Education	1.89	0.93–3.85	1.66	0.82–3.37	0.84	0.36–1.95	0.83	0.37–1.83
Science, Maths, Computing	2.61**	1.85–3.67	1.49*	1.05–2.12	1.81**	1.25–2.63	1.46*	1.06–2.02
Engineering & Manufacturing	2.02**	1.26–3.27	1.34	0.81–2.23	1.25	0.73–2.16	1.31	0.82–2.09
Other	2.89**	1.58–5.27	1.52	0.78–2.98	2.87**	1.50–5.52	1.62	0.87–2.99
*Accommodation (Reference Cluster = Living at Home*								
Away from home	0.91	0.72–1.15	0.92	0.71–1.19	3.05**	2.34–3.98	1.16	0.92–1.46
Female Students								
Cluster Title	Low PA & Smoking		Convenience Food			Alcohol Consumption		
N	575		491			311		
Participant Demographics								
	OR	95% CI	OR		95% CI	OR	95% CI	
Age *(years)*	1.03**	1.01–1.04	0.96**		0.93–0.98	0.99	0.97–1.02	
Field of Study *(Reference Cluster = Health, Welfare & Exercise)*								
Social, Business, Law	1.76**	1.29–2.39	1.60**		1.16–2.20	1.46	1.00–2.13	
Humanities & Arts	1.70**	1.23–2.35	1.19		0.84–1.68	1.33	0.89–1.99	
Education	0.32**	0.19–0.53	0.81		0.54–1.21	0.86	0.55–1.36	
Science, Maths, Computing	1.59**	1.15–2.21	1.28		0.90–1.81	1.07	0.71–1.63	
Engineering & Manufacturing	1.25	0.59–2.66	1.43		0.68–2.99	1.12	0.43–2.88	
Other	1.15	0.73–1.82	0.99		0.60–1.63	0.63	0.32–1.23	
*Accommodation (Reference Cluster = Living at Home)*								
Away from home	1.11	0.90–1.36	0.75**		0.61–0.93	1.72**	1.31–2.26	

Binary logistic Regression: Reference category = Ideal Healthy Cluster (males: *n* = 649; females: *n* = 1147); * = p < 0.05, ** = p < 0.01,   OR = Odds Ratio; NS = not significant; 95% CI = 95% Confidence Interval. Numbers are reduced due to missing data for demographic data

For females, the regression models were significant for all clusters when compared to the ‘Ideal Healthy’ cluster (X^2^(8) = 26.739–83.552, *p* < 0.01; R^2^ = 2.8–6.6%). A one year increase in age increased the likelihood of being categorised in the ‘Low PA & Smoking’ cluster (OR = 1.03, CI = 1.01–1.04), but showed a decreased likelihood of being placed in the ‘Convenience Food’ cluster (OR = 0.96, CI = 0.93–0.81). Females in social, business and law (OR = 1.76, CI = 1.29–2.39), humanities and arts (OR = 1.70, CI = 1.23–2.35), and science maths and computing (OR = 1.59, CI = 1.15–2.21) courses had a higher likelihood of being classified in the ‘Low PA & Smoking cluster’ when comparted to the health, welfare and exercise students. Contrastingly, females studying education courses had a decreased likelihood of being categorised in this cluster (OR = 0.32, CI = 0.19–0.54). Females in social, business and law courses also had a higher likelihood of falling into the ‘Convenience Food’ cluster (OR = 1.60, p < 0.01, CI = 1.16–2.20). Females living away from home had a decreased likelihood of being classified in the ‘Convenience Food’ (OR = 0.75, CI = 0.61–0.93) cluster, but an increased likelihood of being classified in the ‘Alcohol Consumption’ (OR = 1.72, CI = 1.31–2.26) cluster.

## Discussion

To the best of the authors’ knowledge, this is the first study to examine the clustering of HRB based on sex and their associations with students’ characteristics in Irish universities. The results of this study show that HRB cluster in this university population, much like previous research in the general Irish population [17] and university students in other countries [1, 14–16, 35]. Cluster outputs for male and female students were similar with the only difference being that drug use was found to be a prominent behaviour in the male clusters only. The co-existence of behaviours in this population is complex, with engagement in HRB varying in each cluster group. As Noble and colleagues (2015) found, our study identified an ‘Ideal Healthy’ cluster and a number of clusters containing a combination of healthy and risky HRB. For example, the risky clusters included meeting the PAGL combined with higher alcohol consumption in both sexes. Various associations between the individual behaviours have been observed in the research with positive associations noted for PA and alcohol consumption [5], PA and fresh food consumption [42, 43], and an inverse association for PA and smoking [5]. The combination of healthy and risky behaviours may even be explained by a ‘work hard, play hard’ [44] or ‘sensation-seeking lifestyle’ [45] theory. These results help to understand which HRB cluster indefinitely, somewhat or not at all, aiding the creation of interventions that tackle clustered HRB, which have been more effective and less costly in the past [46].

Students’ age, field of study and accommodation type were shown to influence the likelihood of being categorised in a cluster containing risky HRB, as opposed to the desired ‘Ideal Healthy’ cluster. These findings allow us to understand which sub-groups of the university population are more likely to engage in risky HRB helping direct the appropriate interventions to the populations most in need. Increases in age were shown to elevate the likelihood of students being classified in the ‘Low PA & Poor Diet’, ‘Alcohol Consumption’ and ‘Smoking & Drug Use’ clusters in males and in the ‘Low PA & Smoking’ cluster in females. Research has reported a decline in PA as individuals age [47], and increases in alcohol consumption for students in later years of study [7], but is yet to note any association between age and smoking or drug use in this population. A suggestion for this may be that older students have had more years of independent living and exposure to risky HRB, such as smoking and drug usage, which has increased their likelihood of engagement. It may be important to use interventions preventing the initiation of these behaviours during the adolescent and early adult years, with interventions designed to cease engagement in these behaviours more applicable for older students who have had increased exposure to both independent living and risky HRB. In contrast, an increase in age decreased the likelihood of females being classified in the ‘Convenience Food’ cluster, with research showing that age and female sex were positively associated with indicators for healthy dietary habits in a representative sample of Norwegian adults [48].

Cluster members also varied based on their field of study and accommodation type. Descriptive analysis allowed us to observe the proportion of students classified in each cluster, while regression analysis examined the influence of students’ characteristics on the likelihood of being placed in a cluster containing risky HRB. The ‘Ideal Healthy’ cluster contained a significantly higher proportion of health, welfare and exercise related students in males and females. This field of study often contains learning modules that increase the knowledge of exercise and health, which is a known determinant of PA and other HRB [49]. In comparison to the health, welfare and exercise students, these results show that students studying certain fields of study have an increased likelihood of being classified in clusters containing risky HRB. Interfaculty differences have been noted in the past, with the prevalence of smoking and drug use increased in arts, public relations, public administration, and communications courses when compared to degrees containing health, welfare and exercise components [50]. In addition, Valera-Mato and colleagues (2012) found no interfaculty differences for alcohol consumption in 985 Spanish university students (32.6% male), but we have identified this to be increased in males studying social, business and law related, and humanities and arts courses when compared to the health, welfare and exercise students. It is recommended, that all university courses include a module or workshop in their curricula providing information on the risks of engagement in certain behaviours. This can be used to promote healthy behaviours while preventing engagement in risky HRB among all students, and not just those studying health, welfare and exercise related courses.

For accommodation, female students living in their family home had an increased likelihood of being categorised in a cluster containing a higher frequency of convenience food consumption. This contrasts previous findings that students living in their family home display healthy dietary behaviours when compared to those living outside of the family home [22, 50]. Students living in university accommodation are more likely to eat in campus facilities [51], where more fresh foods and healthy options are being offered in recent times. This may have a positive influence on students’ dietary behaviours while living away from home and should continue to be encouraged in university food outlets. Living away from the family home was found to increase the likelihood of both male and female students being categorised in clusters with an increased frequency of alcohol consumption. The association between alcohol consumption and living away from home has been reported [23, 24], with White and colleagues finding (2006) that leaving home was a stronger predictor of increased drinking behaviour than was university attendance. There is a need to publicise the risks of drinking alcohol past moderation among Irish students, especially those living away from home. Unfortunately, a high frequency of alcohol consumption tends to be accepted in Irish students, where it is integrated into the social norms of university life. Successful interventions in the past have altered the beliefs or social norms of students so that high levels of alcohol consumption are not seen as normal behaviour [52]. Norman and colleagues (2017) used theory of planned behaviour-based messages targeting key beliefs about binge drinking in students, three weeks before attending university. These focused on the beliefs that students can have fun without binge drinking, that binge drinking can have a negative impact on studies, and that being a student does not mean you have to binge drink alcohol. Students who received the messages engaged in binge drinking less frequently and had less harmful patterns of alcohol consumption during the first 6 months of university [52]. Overall, understanding the individuals within each cluster allows us to identify students who are at risk and may be potential targets for such health promotion efforts.

This study addresses a topic where limited research has focused on the Irish university population and could be considered the most important strength. The study also employed a data-driven approach to determine behavioural clusters and used empirical measures to minimise subjectivity in deciding the number of clusters. A limitation of this study was that the HRB included in the cluster analysis consisted of self-reported responses and did not assess the quantity (e.g. units of alcohol), only the frequency (excluding the IPAQ-SF). Future studies should look at both the frequency and the quantity of different HRB in this population to gain a greater understanding into the participation levels. Similarly, the dietary behaviour measurement tool presents similar problems, limiting the information gathered. It is advised that future studies look to employ more in-depth measurement tools [53] in order to understand the dietary behaviours of students. The behavioural clusters found in this university sample were determined using exploratory cluster analysis and therefore may not be generalizable to other populations. In addition, if other HRB (e.g. sexual practices) were included, different cluster groups may have arisen, while additional variables (e.g. household income) added to the regression analysis may have altered the results. Lastly, this study was cross-sectional, which means that the data only provides a snapshot of how HRB cluster amongst the population.

Understanding the behaviours of university students’ is important as both the increase of independent living they experience and the multiple stressors of university life create an environment that supports the engagement of risky HRB [4]. Health professionals should take note of how HRB cluster when designing multi-health interventions. For example, the targeting of smoking and low PA levels together in females, as opposed to individually, which can have accumulative health effects and be less costly [46]. Similarly, from investigating students’ field of study, modules promoting healthy behaviours may be beneficial in all fields of study and not just for those studying health, welfare and exercise related courses. In addition, it is recommended to target students based on certain characteristics, such as the inclusion of interventions to prevent or cease high frequencies of alcohol consumption in students living away from their family homes. However, more research is needed to investigate why certain sub-groups of students are highly represented within clusters involving risky health or poor dietary behaviours.

## Abbreviations

AR: Adjusted residual
HRB: Health-related behaviours
IPAQ-SF: International physical activity questionnaire - short form
PA: Physical activity
PAGL: Physical activity guidelines
SASSI: Student Activity and Sport Study Ireland
